# The role of proteasome activators PA28αβ and PA200 in brown adipocyte differentiation and function

**DOI:** 10.3389/fendo.2023.1176733

**Published:** 2023-05-02

**Authors:** Zeynep Koçberber, Nienke Willemsen, Alexander Bartelt

**Affiliations:** ^1^ Institute for Cardiovascular Prevention (IPEK), Ludwig-Maximilians-University Munich, Munich, Germany; ^2^ German Center for Cardiovascular Research, Partner Site Munich Heart Alliance, Ludwig-Maximilians-University Hospital, Munich, Germany; ^3^ Institute for Diabetes and Cancer (IDC), Helmholtz Center Munich, German Research Center for Environmental Health, Neuherberg, Germany; ^4^ Department of Molecular Metabolism and Sabri Ülker Center for Metabolic Research, Harvard T.H. Chan School of Public Health, Boston, MA, United States

**Keywords:** BAT, brown adipocytes, proteostasis, ubiquitin-proteasome-system, PA28αβ, *PA200*, *Psme1*, *Psme4*

## Abstract

**Introduction:**

Brown adipocytes produce heat through non shivering thermogenesis (NST). To adapt to temperature cues, they possess a remarkably dynamic metabolism and undergo substantial cellular remodeling. The proteasome plays a central role in proteostasis and adaptive proteasome activity is required for sustained NST. Proteasome activators (PAs) are a class of proteasome regulators but the role of PAs in brown adipocytes is unknown. Here, we studied the roles of PA28α (encoded by *Psme1*) and PA200 (encoded by *Psme4*) in brown adipocyte differentiation and function.

**Methods:**

We measured gene expression in mouse brown adipose tissue. In cultured brown adipocytes, we silenced *Psme1* and/or *Psme4* expression through siRNA transfection. We then assessed impact on the ubiquitin proteasome system, brown adipocyte differentiation and function.

**Results:**

We found that *Psme1* and *Psme4* are expressed in brown adipocytes in vivo and in vitro. Through silencing of Psme1 and/or Psme4 expression in cultured brown adipocytes, we found that loss of PAs did not impair proteasome assembly or activity, and that PAs were not required for proteostasis in this model. Loss of *Psme1* and/or *Psme4* did not impair brown adipocyte development or activation, suggesting that PAs are neither required for brown adipogenesis nor NST.

**Discussion:**

In summary, we found no role for *Psme1* and *Psme4* in brown adipocyte proteostasis, differentiation, or function. These findings contribute to our basic understanding of proteasome biology and the roles of proteasome activators in brown adipocytes.

## 1 Introduction

Thermogenic adipocytes produce heat through non-shivering thermogenesis (NST). Mammals - especially infants, small rodents, and hibernating animals - rely on NST for appropriate thermoregulation as it complements or substitutes shivering- or muscle-generated thermogenesis (1). The main class of thermogenic adipocytes are brown adipocytes, collectively forming brown adipose tissue (BAT) depots. Additionally, beige adipocytes with similar thermogenic capacity to brown adipocytes reside in white adipose tissue (WAT) (2, 3). The common denominator of these thermogenic adipocytes is their (potential) expression of Uncoupling-protein 1 (*Ucp1*), a fatty acid-activated proton carrier that uncouples the electron transport chain from ATP production, which is an exothermic reaction resulting in heat generation (1, 3). Alternatively to Ucp1-mediated uncoupling, futile cycling of creatine, calcium, and fatty acids also lead to heat production in thermogenic adipocytes (4). In response to cold, the central nervous system initiates the release of norepinephrine (NE), which, through β-adrenergic receptors, acutely leads to lipolysis and Ucp1 activation. Over the years, several other thermogenic mediators have been identified to promote adipose tissue browning and NST (5–7). To fuel NST, BAT consumes large amounts of triglycerides and glucose (8, 9), which is associated with a beneficial metabolic profile in mice and humans (8–11). In addition to acute activation, prolonged stimulation of NST results in BAT hyperplasia, mitochondrial biogenesis, as well as the emergence of beige adipocytes within certain white adipose depots (1, 9). This cellular remodeling of oxidative capacity and lipid metabolism is regulated by a complex network of proteostasis mechanisms, including autophagy and the ubiquitin-proteasome system (UPS) (12–17). BAT activation and tissue remodeling are energy and resource costly processes (1, 8), which is probably why thermogenic adipocytes have developed a remarkably dynamic metabolism, allowing them to shift between dormant and active metabolic state depending on the environmental temperature, diet, and hormonal status.

Protein degradation is a major pillar in maintaining proteostasis and metabolism, and appropriate protein turnover is essential for maintaining healthy thermogenic adipocytes (12–14, 17). The proteasome is important both for quality control of misshapen or damaged proteins as well as for determining the lifespan of proteins, and is, therefore, a key player in shaping the cellular proteome in response to nutritional and other environmental changes (18). The proteasome is a multi-meric complex composed of a 28-unit particle (20S, also called CP), which has a ‘barrel-shaped’ structure with a catalytic core, to which one or two regulatory particles can dock (19). The most common regulatory particle is the 19S particle (also called RP or PA700), which regulates substrate delivery to the 20S in an ubiquitin- and ATP-dependent manner (19, 20). 20S associated with one or two 19S make up the constitutive 26S and 30S proteasome structures, respectively. The abundance of these complexes is partially under the transcriptional control of Nuclear factor erythroid 2-related factor-1 (Nfe2l1, also known as Nrf1 or TCF11) (21). 26S proteasome activity upheld and adapted by the transcriptional activity of Nfe2l1 is required for matching proteasomal activity to the levels of ubiquitinated proteins generated by thermogenic adipocytes during cold and sustained NST (13). In addition to this transcriptional regulation, there are also posttranslational mechanisms regulating proteasome activity. A class of regulatory proteins called proteasome activators (PAs) bind to 20S particles, giving rise to a variety of alternative proteasome complexes, whose functions are less well understood (22, 23). The two cytosolic PAs are PA28αβ (also known as PA28, REG or 11S), and PA200 (also known as Blm10 in yeast). PA28αβ is a heptameric PA composed of four PA28α (encoded by *Psme1*) and three PA28β (encoded by *Psme2*) units (24), and are associated with the immune response and oxidative stress (23, 25, 26). PA200 (encoded by *Psme4*) is a large monomer (circa 200 kDa) that is implicated in the regulation of proteasome activity in the context of DNA repair (23, 27–29). In previous work, we showed that Psme1 expression induces proteasome activity in mice (13). However, the function and importance of PAs for proteasome function and proteostasis in brown adipocyte is currently unknown. As the adaptive regulation of 26S proteasome activity is an essential part of NST and BAT function, we hypothesized that 20S-PAs structures play a role in this process. Here, we systematically investigate the roles of PA28αβ and PA200 in brown adipocytes by manipulating *Psme1* and *Psme4* expression *in vitro*.

## 2 Material and methods

### 2.1 Mice husbandry and tissue collection

All animal experiments were performed with approval of the local authorities (License: ROB-55.2-22532.Vet_02-30-32). Mice were housed in individually ventilated cages at room temperature, with a 12-hour light/dark cycle, and fed chow diet (Sniff) and water ad libitum. For cold exposure, 12-week-old male C57BL/6J mice (purchased from Janvier) were exposed to 4 °C for seven days in a Memmert Climate Chamber HPP750 Life. For tissue collection, the afore-mentioned cold exposed mice and 16-week-old male mice with a C57BL/6J background were injected with a lethal dose of xylazine/ketamine (8/120 mg/kg mouse body weight). For primary cell collection, 6-week-old male C57BL/6J (Janvier) were killed by cervical dislocation. Interscapular and supraclavicular BAT was collected from the animals and freshly used for primary cell isolation.

### 2.2 Primary cell collection and culture

For primary cell culture, the collected BAT was first minced and then digested in DMEM/F-12 (Sigma-Aldrich, supplemented with 1.2 U/mL Dispase (Roche), 1 mg/mL collagenase type 2 (Worthington), 15 mg/mL fatty acid free BSA (Sigma-Aldrich), and 0.1 mg/mL DNase 1 (Roche)) at 37 °C, on a shaker, for 30 minutes. The digestion was stopped by supplementing fetal bovine serum (FBS, Sigma-Aldrich). The suspension was filtered first through a 100 μM filter and then through a 30 μM filter. The stromal vascular fraction (SVF) was plated and cultured in DMEM/F-12 (supplemented with 10% v/v FBS and 1% v/v penicillin/streptomycin (Thermo Fisher Scientific)). The cells were incubated at 37 °C, 5% CO_2_. After reaching confluency, the pre-adipocytes were differentiated into mature brown adipocytes. From day 0 (confluence) to day 2, the cells received DMEM/F-12 supplemented with 1 μM dexamethasone (Sigma-Aldrich), 340 nM insulin (Sigma-Aldrich), 500 μM isobutylmethylxanthine (Sigma-Aldrich), 2 nM triiodothyronine (Sigma-Aldrich), and 1 μM rosiglitazone (Cayman). From day 2 until day 6, the cell received DMEM/F-12 supplemented with 10 nM insulin, 2 nM triiodothyronine, and 1 uM rosiglitazone. The medium was refreshed every other day.

### 2.3 Immortalized cell culture and treatment

The immortalized WT-1 mouse brown preadipocyte cell-line (kindly provided by Brice Emmanueli, University of Copenhagen) was grown in DMEM Glutamax (Thermo Fisher, supplemented with 10% v/v FBS and 1% v/v penicillin/streptomycin). After the pre-adipocytes reached confluency (day 0), their differentiation was induced with induction medium (DMEM Glutamax, supplemented with 860 nM insulin, 1 μM dexamethasone, 1 μM triiodothyronine, 1 μM rosiglitazone, 500 μM 3-isobutyl-1-methylxanthine, and 125 μM indomethacin (Sigma-Aldrich). After 48 hours, the induction medium was changed to differentiation medium (DMEM Glutamax, 1 μM triiodothyronine, 1 μM rosiglitazone). The differentiation medium was refreshed every other day. Cells were fully differentiated after 5-6 days. For target gene RNA inhibition (RNAi), cells received 30 nM SMARTpool silencing RNA (siRNA, Dharmacon) through reverse transfection with Lipofectamine RNAiMAX transfection reagent (Thermo Fisher) according to manufacturer’s protocol. Transfection took place one day before induction (day -1). Cell treatments took place on day 5 of differentiation. Cells were treated with 100 nM Bortezomib (Sellect) for 6 or 24 hours, or 1 μM CL-316,143 (Tocris) for 3 hours, or dimethyl sulfoxide (DMSO) as control. Cells were harvested as pre-adipocytes (day 0), early brown adipocytes (day 3) or mature brown adipocytes (day 5-6). Unless mentioned otherwise, assays were performed on mature (day 5) adipocytes.

### 2.4 Gene expression analysis

RNA was extracted from tissues or cells with NucleoSpin RNA kit (Machery Nagel) according to the manufacturer’s instructions, and RNA concentration was determined with NanoDrop (Thermo Fisher). RNA was synthesized into complementary DNA (cDNA) with Maxima H Master Mix (Thermo Fisher) according to the manufacturer’s instructions. To measure gene expression, we combined 10 µg cDNA and 0.5 µM DNA primers with 5 µL PowerUp SYBR Green Master Mix (Applied Biosystems). To establish gene expression, the cycles thresholds (Ct) were calculated in Quant-Studio 5 RealTime PCR system (Thermo Fisher, standard conditions: 2 min on 50°C, 10 min on 95°C, 40 cycles of 15 s on 95°C, and 1 min on 60°C). Normalized virtual copy numbers were was calculated by normalizing the Cts of experimental genes to the Cts of the housekeeper gene *TATA-box binding protein* (*Tbp*) (Δ Ct). Relative gene expression was calculated by normalizing delta Ct of the experimental groups to the control groups (ΔΔ Ct). The primer sequences are listed in **Supplementary Table 1**.

### 2.5 Protein isolation and analysis

The samples were collected in RIPA buffer (50 mM Tris (Merck, pH = 8), 150 mM NaCl (Merck), 0.1% w/v SDS (Carl Roth), 5 mM EDTA (Merck), and 0.5% w/v sodium-deoxycholate (Sigma–Aldrich)) freshly supplemented with a protease inhibitor (Sigma-Aldrich). Samples were lysed in a tissue lyser and then cells were centrifuged twice and tissue lysates were centrifuged three times for 30 min (4°C, 21,000 *g*), to remove lipids and debris. Protein concentrations were determined with Pierce BCA assay (Thermo Fisher). Per sample, 15-30 µg proteins were denatured with 5% v/v 2-mercaptoethanol (Sigma-Aldrich) for 5 min at 95 °C. The denatured samples were loaded in a Bolt 4-12% Bis-Tris gel (Thermo Fisher). After separation, proteins were transferred onto a 0.2 µm PVDF membrane (Bio-Rad) using the Trans-Blot Turbo™ system (Bio-Rad, 25 V, 1.3 A for 7 min). The membrane was blocked in Roti-Block (Roth) for one hour at room temperature. The membranes were incubated overnight in primary antibody dilutions (1:1000 in Roti-block) at 4 °C. The following primary antibodies were used: β-tubulin (Cell Signaling, 2146), Psmb4 (Santa Cruz, sc-390878), Psmd2 (Santa-Cruz, A-11), Psme1 (Abcam, ab3333), Psme4 (Thermo Fisher, PA1-1961), Nfe2l1 (Cell Signaling, 8052), Ubiquitin/P4D1 (Cell Signaling, 3936), Ucp1 (Abcam, ab10983), Hsp90 (Cell Signaling, 4877), and Proteasome 20S alpha 1 + 2 + 3+5 + 6+7 (Abcam, ab22674). The next day, the membrane was washed with TBS-T (200 mM Tris (Merck), 1.36 mM NaCl (Merck), 0.1% v/v Tween 20 (Sigma)), and incubated in secondary antibody (Santa Cruz) (1:10,000 in Roti-block) for 1h at room-temperature. The membranes were developed with SuperSignal West Pico PLUS Chemiluminescent Substrate (Thermo Fisher) in a Chemidoc MP imager (Bio-Rad). Full-size blot images are displayed in **Supplementary Figure 2**.

### 2.6 Native PAGE: in-gel activity assay and immunoblot

The protocol for Native PAGE in-gel proteasome activity assay and subsequent immunoblotting was previously described in detail (15). Briefly, samples were lysed in OK-lysis buffer (50 mM Tris/HCl, pH = 7.5, 2 mM dithiothreitol, 5 mM MgCl_2_ 10% v/v glycerol, 2 mM ATP, 0.05% v/v Digitonin (Thermo Fisher)), kept on ice for 20 minutes, and centrifuged thrice. 15 µg protein, determined with Bio-RAD Protein Assay Kit II, was loaded in a NuPAGE 3-8% Tris-Acetate gel (Thermo Fisher). The gel was run at a constant voltage of 150 V for four hours. The gel was then incubated in a reaction buffer (50 mM Tris, 1 mM MgCl_2_, 1 mM dithiothreitol) for 30 minutes at 37 °C. The fluorescence signal was measured in ChemiDoc MP. Next, the gel was prepared for protein transfer by 15 minutes incubation in a solubilization buffer (2% w/v SDS, 66 mM Na_2_CO_3_, 1.5% v/v 2-mercaptoethanol. The proteins were transferred to a PVDF membrane by ‘wet’ tank transfer (40 mA, overnight). The immunoblot was further treated as described above (see **2.5**). Full size blot images can be found in **Supplementary Figure 2**.

### 2.7 Viability assay

AquaBlueR (MultiTarget Pharmaceuticals) was used to assess cell viability. Cells were incubated in 1:100 AquaBlueR for four hours at 37 °C. Fluorescence was measured at 540/590 nm (excitation/emission) in a Spark 20M Plate reader (Tecan).

### 2.8 Lysate proteasome activity

Cells were lysed in lysis buffer (40 mM Tris (Merck, pH = 7.2), 50 nM NaCl (Merck), 5 mM MgCl_2_(6H_2_O) (Merck), 10% v/v glycerol (Sigma), 2 mM ATP (Sigma), 2 mM 2-mercaptoethanol (Sigma)). Proteasome Activity Fluorometric Assay II Kit (UBPBio, J41110) was used according to the manufacturer’s instructions to measure trypsin-like (T-L), chymotrypsin-like (CT-L), and caspase-like (C-L) proteasome activity. The fluorescent signaling was measured in the plate reader and the results were normalized to DNA with the Quant-iT PicoGreen dsDNA assay kit (Invitrogen, p7589), according to manufacturer’s instructions.

### 2.9 Oil-Red-O (ORO) staining

ORO staining was used to measure lipid content. Cells were washed with cold DPBS (Gibco), fixed in zinc formalin solution (Merck) for 15 minutes at room-temperature and again washed with 2-propanol (Merck). The cells were dried, incubated in 60% v/v ORO (Sigma) for 10 minutes at room-temperature followed by washing with water for 3-4 times. A picture of the plate was taken to visualize the lipid content. To measure absorption, ORO was eluted in 100% 2-propanol, and measured in the plate reader.

### 2.10 Free fatty acid release assay

To measure lipolysis in cell culture supernatants, Free Glycerol Reagent (Sigma F6428) and Glycerol standard solution (Sigma G7793) were used. Cell culture medium was collected to measure free glycerol content and the experiment was performed according to the manufacturer’s instructions.

### 2.11 Extracellular flux analysis (seahorse)

Oxygen consumption rate (OCR) was measured in a Seahorse XFe24 Analyzer (Agilent) and the assays performed as previously described (30). Briefly, we performed a Seahorse Cell Mito Stress Test (Agilent) largely according to manufacturer’s instructions, but with the addition of a NE (1 μM) injection. There was no addition of BSA to the medium at any point. Two days before the assay, 20,000 adipocytes were seeded per well. During the assay, cells were consecutively treated with NE, oligomycin (1 μM), FCCP (4 μM), and Rotenone/Antimycin A (both 0.5 μM). Oxygen consumption was measured in intervals of 3 minutes. The results were normalized to total DNA levels which were measured with CyQuant Cell Proliferation Assay (C7026, Invitrogen) according to manufacturer’s instructions. NE-induced respiration was calculated by subtracting maximum baseline OCR from maximum NE-induced OCR. Coupled respiration is baseline OCR minus OCR after oligomycin. Uncoupled respiration is OCR after oligomycin minus OCR after Rot/AA injection. Maximum respiration was calculated by subtracting minimum OCR, measured after Rot/AA injection, from maximum OCR, measured after FCCP injection.

### 2.12 Data analysis and visualization

All data was analyzed with Excel and GraphPad Prism. The raw data from the Seahorse was analyzed with Wave software (Agilent). Immunoblots were quantified with ImageLab (Bio-Rad). Data was visualized in GraphPad Prism. If not otherwise specified, data is represented as mean ± standard error of the mean (SEM). (Multiple) Student’s t-test (with Bonferroni post-hoc test) was used to compare two groups with one variable. One-way ANOVA with Tukey post-hoc test was used to compare three groups with one variable. Two-ANOVA with Tukey post-hoc test was used to compare four groups with two different variables, i.e. for the double siRNA transfection experiments. Three-way ANOVA with Dunett’s post-hoc test was used to compare more than four groups with more than two different variables, i.e. for the double siRNA transfection plus treatment experiments. P-values lower than 0.05 were considered significant. If groups are significantly different from each other, this is indicated in graphs either with an asterisk (*) or with different letters (a, b). If the same letter is used or if nothing is indicated, the groups are statistically indifferent from each other. The graphics were made in Biorender.com.

## 3 Results

### 3.1 Proteasome activators are expressed in brown adipocytes

**Figure 1:**
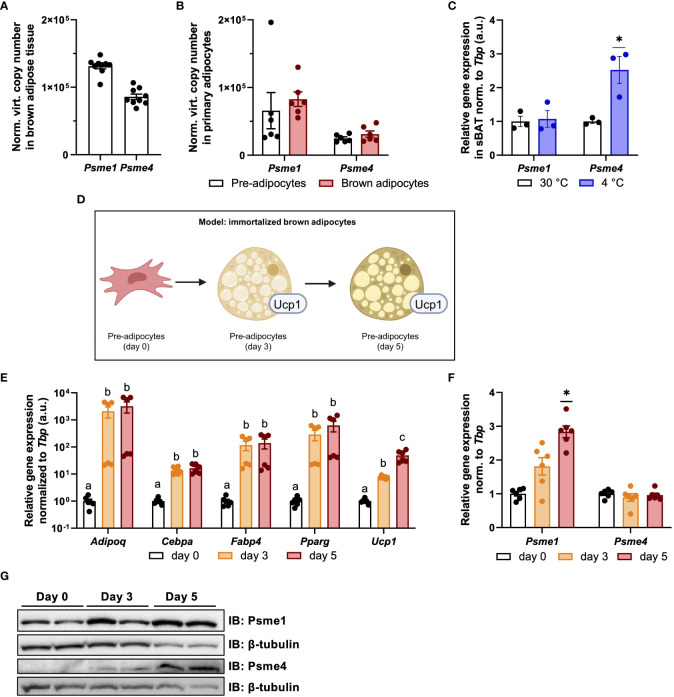
Psme1 and Psme4 expression in brown fat and brown adipocytes. **(A, B)** Normalized gene expression of *Psme1* and *Psme4* in **(A)** brown adipose tissue (BAT) (n = 9 biological replicates), and **(B)** primary brown pre-adipocytes and mature adipocytes (n = 6 biological replicates). **(C)** Relative gene expression of Psme1/Psme4 in BAT after cold exposure (1 week at 4 °C) (n = 3 biological replicates). **(D)** Summary of experimental model made in Biorender.com. **(E)** Relative gene expression of *Adipoq, Cebpa, Fabp4, Pparg* and *Ucp1* in immortalized brown adipocytes in different stages of development: pre-adipocytes (day 0), early adipocytes (day 3), and mature adipocytes (day 5). (n = 6 measurements pooled from two independent experiments). **(F)** Relative gene expression of *Psme1* and *Psme4* in immortalized brown adipocytes in different stages of development: pre-adipocytes (day-0), early adipocytes (day 3), and mature adipocytes (day 5). (n = 6 measurements pooled from two independent experiments). **(G)** Representative immunoblots showing Psme1, Psme4 and β-tubulin. Data are represented as mean ± SEM. Data are significant if *P* < 0.05, which is indicated with an asterisk (*) or by different letters (a, b).

The remodeling of the constitutive proteasome is an essential component of brown adipocyte adaptation to sustained activation (13), but PAs are not part of the 26S/30S constitutive proteasome. Instead, they form hybrid shapes with the 20S core particle (22). It is unknown if PS are expressed in brown adipocytes, if proteasome hybrids are present in and if so, what their roles are in brown adipocyte biology. Therefore, we first assessed the levels of PAs in BAT ex vivo and found that both *Psme1* and *Psme4* were abundantly expressed in the tissue (**Figure 1A**). Next, we measured *Psme1* and *Psme4* gene expression before and after brown adipocyte differentiation in primary cells obtained from the interscapular brown adipose tissue derived stromal fraction (SVF). Both genes were expressed in pre-adipocytes and mature brown adipocytes (**Figure 1B**). Following this, we determined if these genes were differentially expressed during states of BAT inactivity and BAT induction. Mice were exposed to either thermoneutrality (30°C) or to cold (4°C) for one week. Thermoneutrality initiates BAT whitening and cold activates NST and promotes tissue browning. While *Psme1* and *Psme4* were expressed under both conditions, *Psme4* expression was highest in cold-exposed mice (**Figure 1C**). For the remainder of the experiments in this manuscript, we used an immortalized mouse brown pre-adipocyte cell line (Simplified model in **Figure 1D**). These pre-adipocytes differentiate into mature brown adipocytes within six days. After three days of induction, there was a marked increase in expression of the adipogenesis markers *Adipoq*, *Cebpa*, *Fabp4*, and *Pparg*, as well as brown adipocyte marker *Ucp1* (**Figure 1E**). In these cells, we measured both gene expression and protein levels of Psme1 and Psme4 during different stages of cell differentiation. We found increased gene expression of *Psme1* and increased protein level of Psme1 during differentiation (**Figures 1F, G**). In contrast, *Psme4* expression did not change during differentiation, but protein Psme4 was only detectable in early and mature brown adipocytes, and not in pre-adipocytes. (**Figures 1F, G**). In summary, Psme1 and Psme4 are constitutively expressed in both pre-adipocytes and brown adipocytes, and Psme1 expression is induced during differentiation whilst Psme4 expression is induced with cold-induced activation *in vivo*.

### 3.2 The effect of loss of PAs on viability, stress, and inflammation

**Figure 2:**
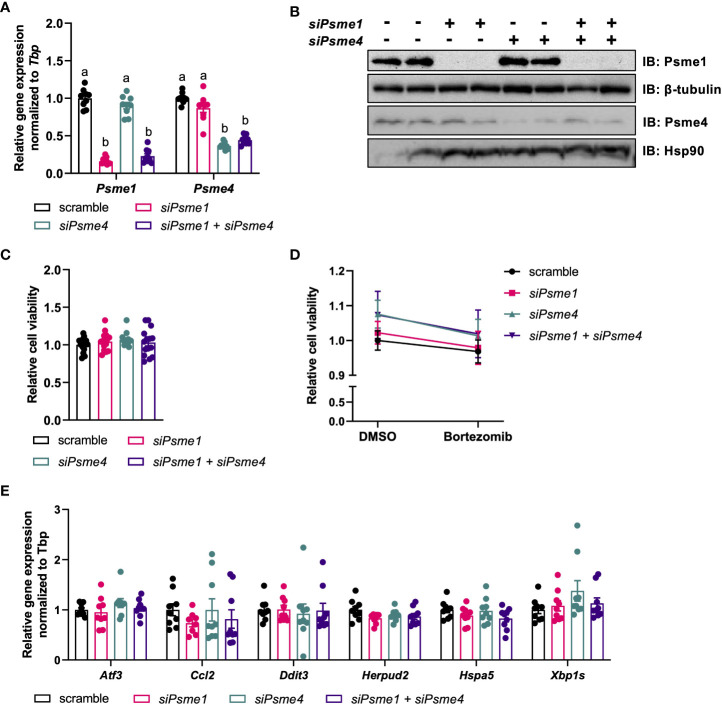
Silencing of Psme1 and/or Psme4 had no effect on viability. **(A)** Relative gene expression of *Psme1* and *Psme4* after transfection with si*Psme1* and/or si*Psme4*. **(B)** Representative immunoblots showing Psme1, Psme4, β-tubulin and Hsp90. **(C)** Relative cell viability after transfection measured with AquaBlueR (n = 15 measurements pooled from three independent experiments). **(D)** Relative survival after treatment with DMSO or Bortezomib (100 nM for 16 h) measured with AquaBlueR. (Mean of n = 15 measurements pooled from three independent experiments). **(E)** Relative gene expression of inflammation and stress markers. Genes measured are *Atf3*, *Ccl2*, *Ddit3*, *Herpud2*, *Hspa5*, *Xbp1s.* Graphs show. Unless indicated otherwise, n = 9 measurements pooled from three independent experiments. Data are represented as mean ± SEM. Data are significant if *P* < 0.05, which is indicated with an asterisk (*) or by different letters (a, b).

As the PAs Psme1 and Psme4 were present and regulated in brown adipocytes, we hypothesized that they could play a role in brown adipocyte function. In order to study their roles, we silenced *Psme1* and *Psme4* gene expression in brown adipocytes. We transfected cells before differentiation (day -1) with either *Psme1* siRNA, *Psme4* siRNA, or the combination of both. The knockdown successfully led to lower levels of gene expression and kept gene expression low even after differentiation (**Figure 2A**). This translated into an almost complete ablation of protein levels for both PAs (**Figure 2B**). First, we checked if silencing of PAs resulted in any impaired cell viability and found no effects of *Psme1* and/or *Psme4* gene silencing (**Figure 2C**). Additionally, we treated the cells with the chemical proteasome inhibitor bortezomib to measure bortezomib-induced cell death, as impairment of the proteasome or its regulation sensitizes cells to treatment with proteasome inhibitors (21, 31). However, loss of Psme1 or Psme4 did not amplify bortezomib-induced cell death (**Figure 2D**). Finally, loss of PAs did not alter gene expression of *Ccl2*, a surrogate marker of adipocyte inflammation, nor that of *Atf3, Xbp1s, Herpud2*, or *Hspa5*, all surrogate markers of protein folding stress (**Figure 2E**) or in the transcription levels of the apoptosis marker *Ddit3* (**Figure 2E**). Overall, loss of Psme1 and or Psme4 did neither cause or enhance bortezomib-induced cell death nor provoke an overt stress response in the cells.

### 3.3 Loss of PAs does not impair UPS in brown adipocytes

**Figure 3:**
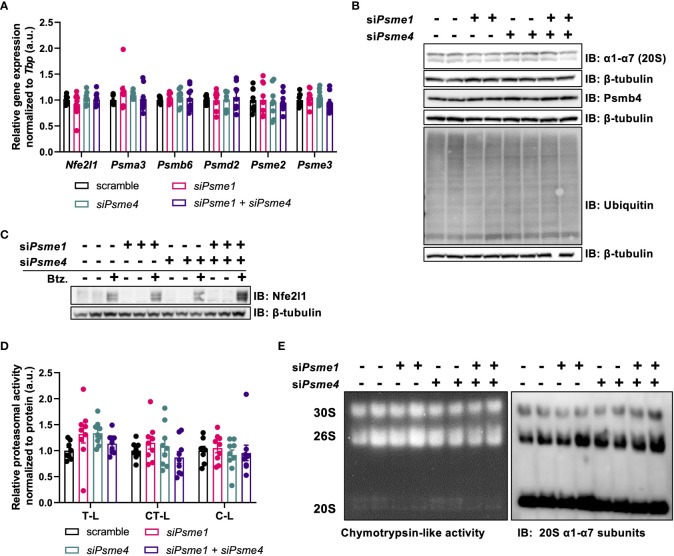
The effects of Psme1/Psme4 knockdown on proteasome activity. **(A)** Relative gene expression of *Nfe2l1* and proteasome subunits *Psma3*, *Psmb6*, *Psmd2*, *Psme2*, and *Psme3*, in brown adipocytes after transfection with si*Psme1* and/or si*Psme4*. **(B)** Representative immunoblots with protein levels of Psmb4, α1-α7, ubiquitin and β-tubulin. **(C)** Representative immunoblots with protein levels of Nfe2l1 and β-tubulin after treating cells with DMSO or Bortezomib (100 nM, 6 h). **(D)** Proteasome activity of chymotrypsin-like (CT-L), trypsin-like (T-L) and caspase-like (C-L) activity. **(E)** Representative native PAGE with in-gel CT-L-activity and immunoblot with α1-α7 (20S) staining. Unless indicated otherwise, n = 9 measurements pooled from three independent experiments. Data are represented as mean ± SEM. Data are significant if *P* < 0.05, which is indicated with an asterisk (*).

PAs bind to 20S core particles, forming hybrid proteasomes, and, thus, PAs are implicated in regulating proteasome activity and substrate selection. To determine if manipulation of PAs impacts brown adipocyte proteostasis, we checked if *Psme1* and/or *Psme4* knockdown affected gene expression of proteasome transcription factor *Nfe2l1* or other proteasome subunits. We measured expression of several proteasome subunits to cover the different parts of the proteasome: *Psma3* and *Psmb6* as representative units of the 20S, *Psmd2* as part of the 19S regulatory particle, *Psme2* as part of the PA28αβ complex, and *Psme3* for the nuclear PA28γ. We found no differences in any of these tested transcripts (**Figure 3A**). Correspondingly, we found no changes in protein levels of the seven α-subunits, ranging from Psma1 to Psma7, and of Psmb4 comparing controls cells and cells with *Psme1* and/or *Psme4* knockdown (**Figure 3B**). Additionally, both baseline and bortezomib-induced Nfe2l1 protein levels were unchanged, and both full-length and short-length forms were present in the brown adipocytes (**Figure 3C**). Global ubiquitin levels, as a marker for proteostatic stress and modulation of UPS, were similar between control and experimental groups (**Figure 3B**). To directly measure proteasome activity, we used two distinct methods. For the first approach, we measured trypsin-like, chymotrypsin-like, and caspase-like activity in whole-cell lysates after gene knockdown (**Figure 3D**). For the second approach, we loaded non-denatured proteins in a Native PAGE. This allowed us to visualize the 20S, 26S and 30S proteasome with in-gel proteasome activity and then subsequently quantify protein levels by immunoblotting. In line with the unchanged ubiquitin levels, we also found no impairment in proteasome activity, neither in in-gel chymotrypsin-like activity nor in α1-α7 protein levels (**Figure 3E**). We also investigated if stressing proteostasis with proteasome inhibitor bortezomib under knock-down conditions would result in altered respiration. While bortezomib treatment impaired mitochondrial respiration, this effect was not affected by silencing of Psme1 or Psme4 (**Supplementary Figures 1A, B**). Overall, this set of experiments demonstrated that Psme1 and Psme4 are dispensable for proteasome availability and function in brown adipocytes.

### 3.4 Loss of PAs does not affect adipogenesis or brown adipocyte function

**Figure 4:**
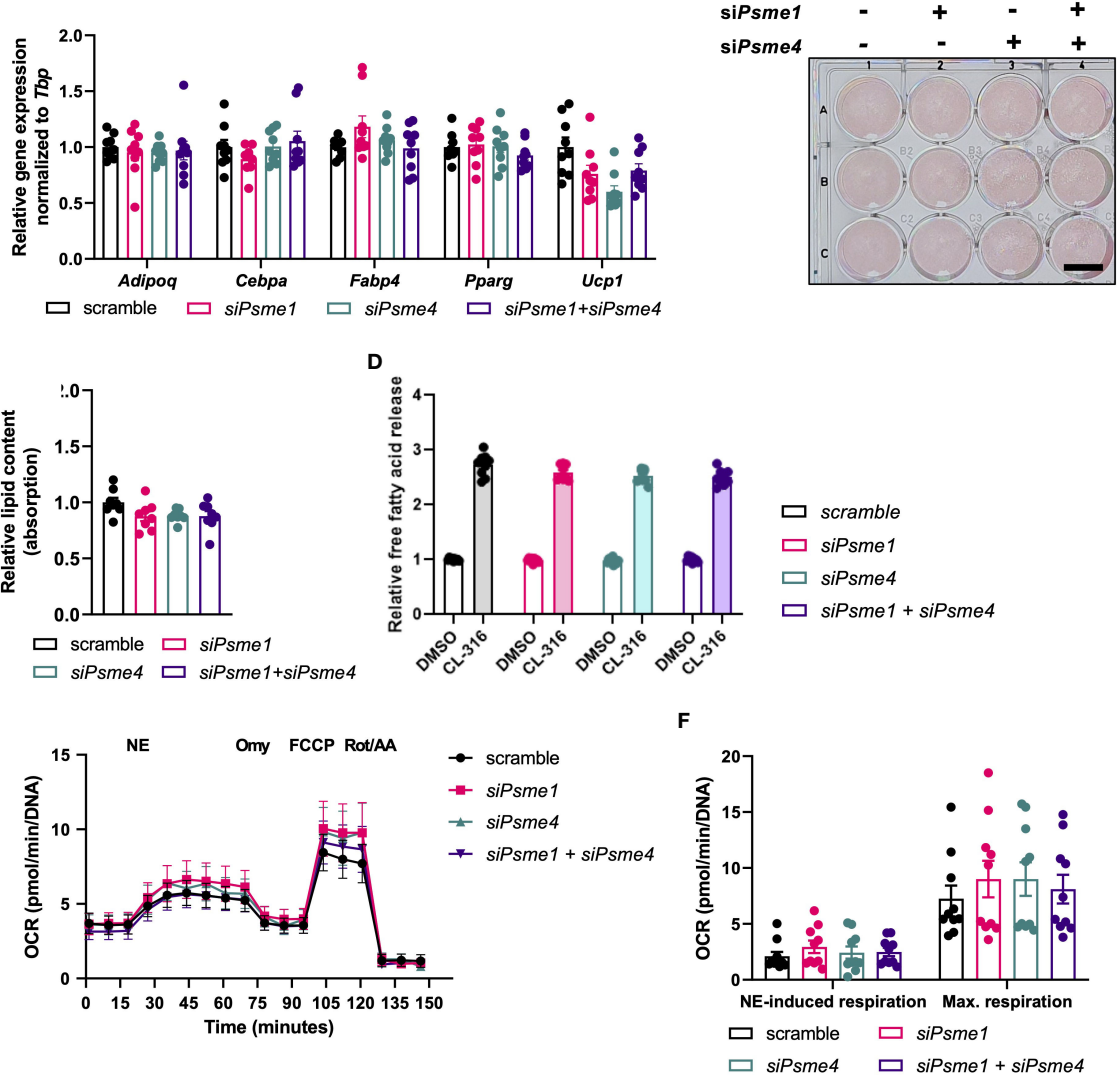
Loss of Psme1/Psme4 did not impair adipocyte function. **(A)** Relative gene expression of adipocyte markers in brown adipocytes after transfection with si*Psme1* and/or si*Psme4*. Genes: measured: *Adipoq, Cebpa, Fabp4, Pparg* and *Ucp1*. **(B)** Representative Oil-Red-O staining and **(C)** relative lipid content measured as absorption. Scale bar in image equals 1 cm. **(D)** Relative free fatty acid levels in medium after cell treatment with DMSO or CL-316,243 (CL-316) (1 μM, 3 hours). **(E, F)** Oxygen consumption rate (OCR) during NE treatment and mitochondrial stress test, normalized to DNA levels (n = 8 measurements pooled from two independent experiments). NE: norepinephrine, Omy: oligomycin, FCCP: carbony cyanide p-trifluoro-methoxyphenyl hydrazone, Rot/AA: rotenone/antimycin A Unless indicated otherwise, n = 9 measurements pooled from three independent experiments. Data are represented as mean ± SEM. Data are significant if *P* < 0.05, which is indicated with an asterisk (*).

Finally, to determine the effect of PAs on brown adipocyte-specific biology, we studied the effects of loss of PAs on brown adipocyte development and function. *Psme1* and/or *Psme4* knockdown did not affect expression of the adipocyte markers *Adipoq*, *Cebpa, Fabp4*, and *Pparg* (**Figure 4A**). However, there was a non-significant trend for lower *Ucp1* mRNA expression in cells with si*Psme4* (**Figure 4A**). Next, lipid content was measured as an indicator for net adipogenesis and lipogenesis. ORO staining showed that lipid content was not lower in the knock-down groups compared to the control groups (**Figures 4B, C**). As brown adipocytes induce lipolysis to fuel heat production (3), we measured cell culture supernatant glycerol levels before and after stimulation with the β3-adrenergic agonist CL-316,243. The glycerol release assay showed no effects upon loss of Psme1 and/or Psme4 (**Figure 4D**). Finally, we measured oxygen consumption rate (OCR) to measure sympathetic response and global cellular respiration. NE-induced respiration was used to measure cellular NST-capacity *in vitro*. NE-induced respiration was not different between groups, nor was maximum respiratory capacity, suggesting that there were no significant changes in mitochondrial abundance and health (**Figures 4E, F**). Even stressing the system with Bortezomib treatment did not differentially affect knock-down groups compared to the control group (**Supplementary Figure 1A, B**). Taking these results together, we found no evidence that loss of PAs impaired adipocyte function.

## 4 Discussion

Brown adipocytes undergo cellular remodeling during thermogenic activation (1), and in order to sustain NST, an appropriate protein turnover is required (14). Although the role of the constitutive 26S proteasome in BAT and NST has been studied previously (13), the role of other proteasome types remains elusive. In this study, we investigated the roles of Psme1/PA28α and Psme4/PA200 in brown adipocyte differentiation and function. We found that *Psme1* and *Psme4* were constitutively expressed in brown pre-adipocytes and mature adipocytes, which alludes to a significant role in brown adipocyte proteostasis. However, in our model of cultured adipocytes, loss of Psme1 and/or Psme4 protein by RNAi did not affect viability or led to a marked stress response in the cells. Moreover, even though we saw minor effects on proteasome activity, UPS and proteostasis remained functional. Finally, neither differentiation nor activation of brown adipocytes was impaired after silencing of *Psme1* and/or *Psme4*. We conclude that in our experimental settings, Psme1 and Psme4 are dispensable for cultured brown adipocytes.

There are limitations to our model and approach that should be noted. Firstly, the experiments were performed in an immortalized brown adipocyte cell line, which cannot mimic the biological complexity and natural regulation of BAT activation or remodeling observed *in vivo* in mice or humans. An adipocyte-specific transgenic deletion mouse model could provide insight into the physiological roles of PAs, but these models have not yet been established. This cell model allowed for basic study of the role of PAs in adipocytes, but did not characterize PAs under different physiological conditions, e.g. nutrient deprivation, inflammation, nor did it take into account cell-cell interactions or systemic effects. Secondly, we limited our study to Psme1 and Psme4, leaving the other proteasome activators subunits Psme2 and Psme3 out of the scope of this study. Admittedly, as PA28αβ consists of Psme1 and Psme2 subunits, it is possible that sole loss of Psme1 would result in an alternative PA28 form with residual activity. However, this PA-variant is thought to be less stable and active, and it is unknown if there is a physiological relevance (24). Furthermore, there could also be compensation mechanism through Psme3 activation, but as Psme3 is a nuclear PA instead of cytosolic, we estimated this chance as low (22). Finally, we used siRNA to knockdown gene expression and this method does not completely ablate protein levels. Even though we observed marked near to complete loss of protein for both Psme1 and Psme4, it is possible that a remaining low expression of *Psme1*/*Psme4* was sufficient to sustain a residual activity. However, we have previously shown that the same experimental strategy resulted in efficient ablation of 20S subunit Psmb4, which disrupted proteostasis, adipocyte differentiation, and thermogenesis (30). In addition, a separate study showed that siRNA-mediated manipulation of Psme4 affected myofibroblast differentiation (32). This indicates that the experimental RNAi strategy is capable of targeting both constitute and adaptive proteasome subunits and investigate their role for brown adipocyte differentiation and activity.

Regardless, the roles of Psme1 and Psme4 in regulating proteasome function and protein degradation are not well-established. Based on their structures, both PA28αβ and PA200 are thought to stimulate the insertion of unfolded proteins or peptides into the 20S proteasomes (22, 23). Also, PA28αβ is associated with the immunoproteasome, a specific type of proteasome that specifically degrades proteins for antigen-presentation (25, 26). However, mice lacking Psme1/Psme2 display no growth abnormalities or obvious health problems (33). In a study with triple-knockout mice, ablation of Psme1, Psme2 and nuclear Psme3/PA28γ (gene: *Psme3*), the researchers found reduced proteasome activity and exacerbated high-fat diet-induced hepatic dysfunction (34), even though the cause of this metabolic phenotype remains unclear. Also, the function of Psme4 is still being debated, but it is associated with the process of DNA repair (27, 28) and was shown to play a role in myofibroblast differentiation (32). Whole-body Psme4 knock-out did not result in an overt phenotype, but showed impaired spermatogenesis and infertility (35, 36). These studies with whole-body knock-out mouse models did not investigate or report any BAT or NST phenotype (32–36), leaving it as an open question if they participate in adipocyte biology *in vivo*. Although we found that Psme1 and Psme4 were not required in brown adipocytes *in vitro* under standard conditions, they may play a role in specific cellular stress responses. Based on its implication of immunoproteasome regulation, Psme1 may play a role in the immune response of the adipocyte, but the role of immunoproteasome formation in adipocytes is unknown, too. Alternatively, Psme1 and or Psme4 could be recruited in response to specific stressors, for example in the adaptive proteasome response against oxidative stress, proteasome autophagy or ferroptosis (37). Interestingly, it was observed that overexpression of Psme1 enhances proteasome activity in obese mouse models when proteasome function was compromised (13). Further scrutiny of the PAs in different contexts will contribute to our understanding of their functions and mechanisms and should determine if and how PAs play a role in adipocytes *in vivo*. In conclusion, our data reveal that, even though expressed high robust levels, Psme1 and Psme4 are dispensable for proteostasis, adipogenesis, and thermogenesis in cultured brown adipocytes.

## Data availability statement

The raw data supporting the conclusions of this article will be made available by the authors, without undue reservation.

## Ethics statement

The animal study was reviewed and approved by Regierung von Oberbayern (ROB) 55.2 22532.Vet_02 30 32.

## Author contributions

AB and NW designed and supervised the study. ZK and NW performed the experiments, analyzed the data, and prepared the figures. AB supervised the study and analyzed the data. The authors wrote the manuscript together. All authors contributed to the article and approved the submitted version.

## Supplementary material

Supplementary Figure 1
**(A)** Relative maximum respiratory capacity of Bortezomib treated cells compared to DMSO treated cells. Treatment was either DMSO or 100 nM Bortezomib for 16 hours pre-ceding the mitochondrial stress test. Maximum respiratory capacity is maximum OCR measured after FCCP treatment minus OCR after Rot/A treatment, before DNA normalization (n = 4). **(B)** Oxygen consumption rate (OCR) from mitochondrial stress test, normalized to DNA levels (n = 4). Max. Resp. = Maximum Respiratory capacity. Data are represented as mean ± SEM. Data are significant if *P* < 0.05, which is indicated with an asterisk (*).

Supplementary Figure 2 Uncropped pictures from immunoblots.
