# Supplementary material for: The role of proteasome activators PA28αβ and PA200 in brown adipocyte differentiation and function
Source: Front Endocrinol (Lausanne). 2023 May 2;14:1176733. doi: 10.3389/fendo.2023.1176733 (PMC10187037; doi:10.3389/fendo.2023.1176733)
Supplement: Supplementary file 1 [file Table_1.docx]

| **Primer** | **Forward sequence (5’ to 3’)** | **Reverse sequence (5’ to 3’)** |
| --- | --- | --- |
| Adipoq | GGAGAGAAAGGAGATGCAGGT | CTTTCCTGCCAGGGGTTC |
| Atf3 | GAGGATTTTGCTAACCTGACACC | TTGACGGTAACTGACTCCAGC |
| Ccl2 | TTAAAAACCTGGATCGGAACCAA | GCATTAGCTTCAGATTTACGGGT |
| Cebpa | AAACAACGCAACGTGGAGA | GCGGTCATTGTCACTGGTC |
| Ddit3 | CTGGAAGCCTGGTATGAGGAT | CAGGGTCAAGAGTAGTGAAGGT |
| Fabp4 | GGATGGAAAGTCGACCACAA | TGGAAGTCACGCCTTTCATA |
| Herpud2 | ATGGACCAAAGTGGGATGGAG | TCAATGGTTTGCTAGGGTACAC |
| Hspa5 | TCATCGGACGCACTTGGAA | CAACCACCTTGAATGGCAAGA |
| Nfe2l1 | GACAAGATCATCAACCTGCCTGTAG | GCTCACTTCCTCCGGTCCTTTG |
| Pparg | TCGCTGATGCACTGCCTATG | GAGAGGTCCACAGAGCTGATT |
| Psma3 | GAAGCAGAGAAATATGCCAAGG | GCAACTGTTACAGAAATGTAAACCA |
| Psmb6 | GAAAACCGGGAAGTCTCCAC | CTCGATTGGCGATGTAGGAC |
| Psmd2 | AATGGGAGATTCCAAGTCCA | TGACATCTCCATTGCAGGAC |
| Psme1 | ATAATTTTGGCGTGGCTGTC | TGGAGATCTGCGTGTGGA |
| Psme2 | gggtggcaattcaggaga | ctacagcgtcccctcgttc |
| Psme3 | CACTGTCACAGAGATTGATGAGAA | GGATCATGTCATGGAGAGTGAC |
| Psme4 | CCTCACACAATGTTCCAAAGAC | GAAACAGAAAAGTTAAAGACCTTCTGA |
| Tbp | AGAACAATCCAGACTAGCAGCA | GGGAACTTCACATCACAGCTC |
| Ucp1 | AGGCTTCCAGTACCATTAGGT | CTGAGTGAGGCAAAGCTGATTT |
| Xbp1s | GGTCTGCTGAGTCCGCAGCAGG | AGGCTTGGTGTATACATGG |

**Supplementary table 1: Primers used for qPCR.**
