# Supplementary figures and images for: The role of proteasome activators PA28αβ and PA200 in brown adipocyte differentiation and function
Source: Front Endocrinol (Lausanne). 2023 May 2;14:1176733. doi: 10.3389/fendo.2023.1176733 (PMC10187037; doi:10.3389/fendo.2023.1176733)

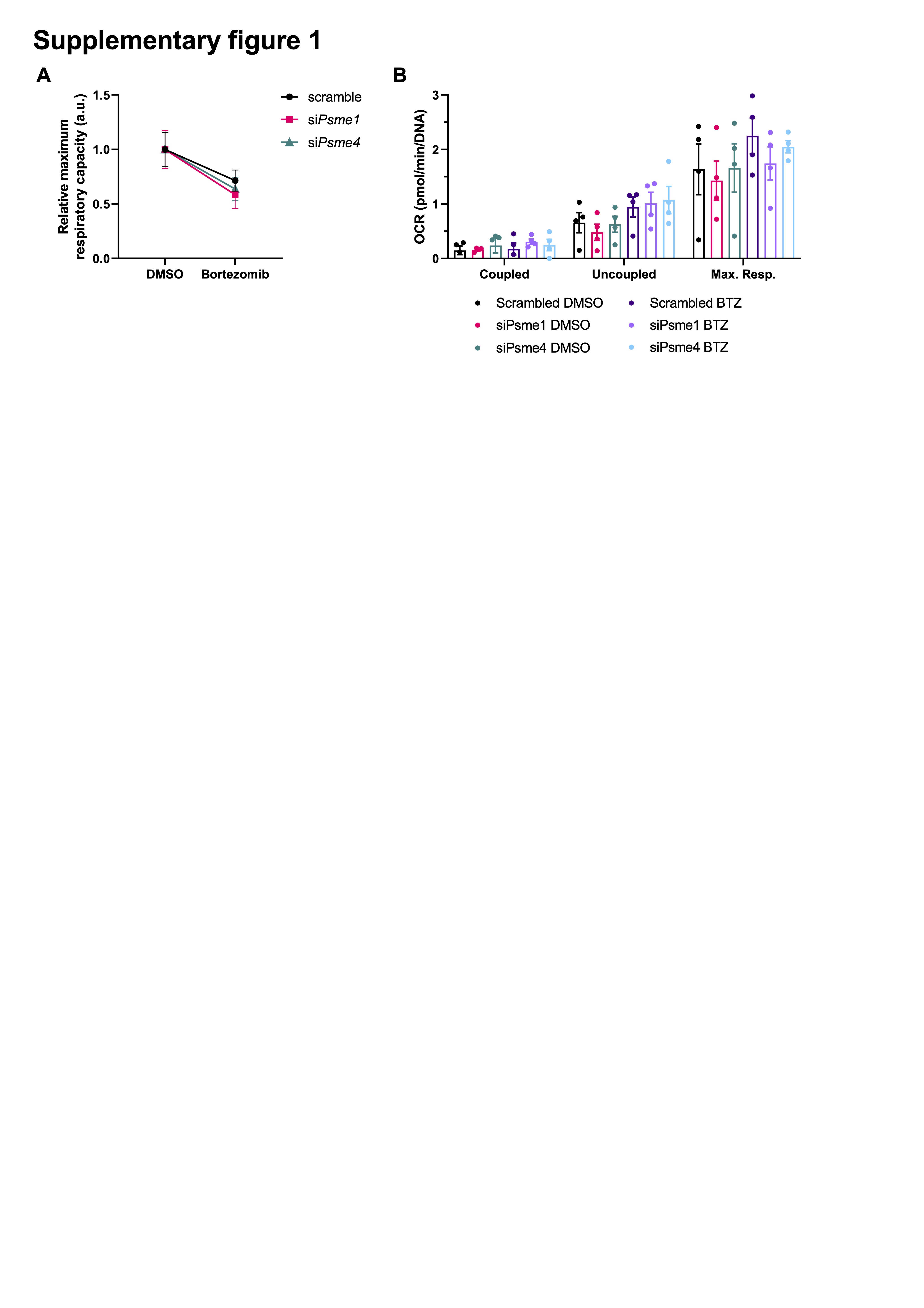

Supplement: Supplementary Figure 1 — (A) Relative maximum respiratory capacity of Bortezomib treated cells compared to DMSO treated cells. Treatment was either DMSO or 100 nM Bortezomib for 16 hours pre-ceding the mitochondrial stress test. Maximum respiratory capacity is maximum OCR measured after FCCP treatment minus OCR after Rot/A treatment, before DNA normalization (n = 4). (B) Oxygen consumption rate (OCR) from mitochondrial stress test, normalized to DNA levels (n = 4). Max. Resp. = Maximum Respiratory capacity. Data are represented as mean ± SEM. Data are significant if P < 0.05, which is indicated with an asterisk (*). [file Image_1.jpeg]

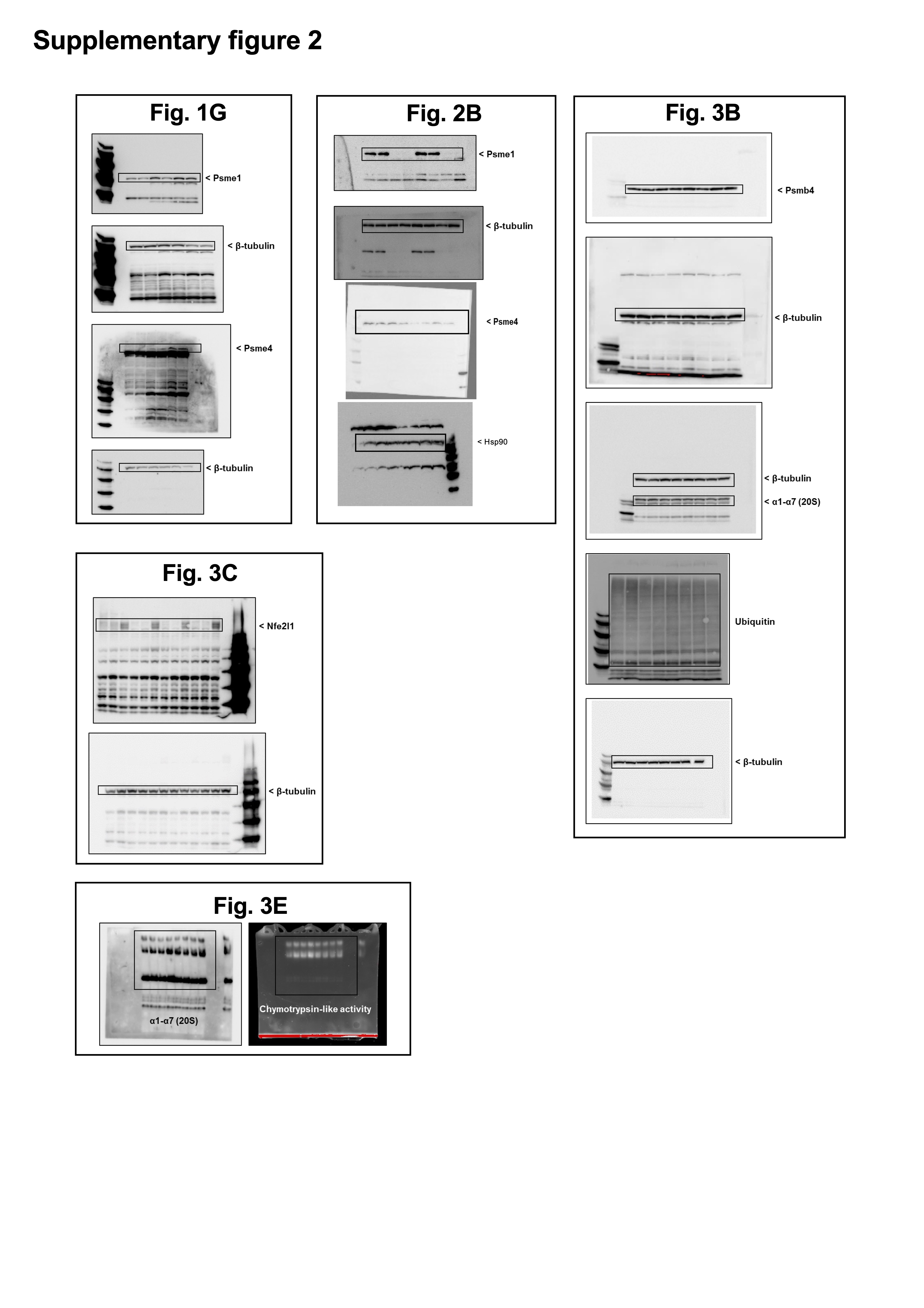

Supplement: Supplementary Figure 2 — Uncropped pictures from immunoblots. [file Image_2.jpeg]
